# Clinical impact of early bronchoscopy in mechanically ventilated patients with aspiration pneumonia

**DOI:** 10.1186/2197-425X-3-S1-A388

**Published:** 2015-10-01

**Authors:** S Park, Y-J Cho, YJ Lee

**Affiliations:** Sheikh Khalifa Specialty Hospital, RAK, United Arab Emirates; Division of Pulmonary and Critical Care Medicine, Seoul National University Bundang Hospital, Bundang, Korea, Republic of Korea

## Background

A handful of studies have reported that the proper timing of bronchoscopy influences the clinical outcome in mechanically ventilated patients with aspiration pneumonia.

## Methods

Mechanically ventilated patients with aspiration pneumonia who received bronchoscopy in a medical intensive care unit at tertiary hospital from March 2003 to December 2013 were retrospectively reviewed. By definition, patients who underwent bronchoscopy within 24 hours after intubation were categorized as the early bronchoscopy group. We compared demographics, clinical parameters, and outcomes, including mortality, between the two groups.

## Results

182 patients were included. Compared to the late bronchoscopy group, the early bronchoscopy group (n = 93) showed no significant differences in demographic features, including acute physiology and chronic health evaluation II scores. The early bronchoscopy group showed significantly lower in-ICU mortality and 90-day mortality (in-ICU: 6.5% vs. 26.1%; 90-day: 10.8 vs. 33.0%) regardless of the appropriateness of the initial empirical antibiotics. In addition, their sequential organ failure assessment score on day 7 tended to decrease more rapidly. The early timing of bronchoscopy was associated with a lower 90-day mortality in multivariate analysis (odds ratio: 0.415; 95% confidence interval 0.183-0.942).

## Conclusions

Early bronchoscopy could benefit the clinical outcomes of mechanically ventilated patients with aspiration pneumonia.

